# Temporal attention affects contrast response function by response gain

**DOI:** 10.3389/fnhum.2022.1020260

**Published:** 2023-01-25

**Authors:** Chengxu Jing, Hongyuan Jin, Wenxia Li, Zhouhao Wu, Yao Chen, Dan Huang

**Affiliations:** ^1^School of Automation and Electronic Information, Sichuan University of Science and Engineering, Sichuan, China; ^2^Artificial Intelligence Key Laboratory of Sichuan Province, Sichuan University of Science and Engineering, Sichuan, China; ^3^School of Biomedical Engineering, Shanghai Jiao Tong University, Shanghai, China

**Keywords:** temporal attention, cue, response gain, contrast response, psychophysics

## Abstract

Orienting attention to a specific point in time has been shown to improve the contrast sensitivity at the attended time point and impair it earlier or later. This phenomenon could be explained by temporal attention increasing the effective contrast of the target presented at the attended time point which leads to changes in contrast psychometric function by contrast gain. Another explanation is that temporal attention multiplicatively amplifies the amplitude of behavioral or neural response to contrast, resulting in alterations in contrast psychometric function by response gain. To explore the underlying mechanism, we adopted a temporal cueing orientation discrimination task using audio pre-cues composed of different frequency components to induce different attentional allocations in the time domain and targets of various contrast intensities to measure contrast psychometric functions. Obtained psychometric functions for contrast sensitivity were fitted for different conditions with discrepant attentional states in time. We found that temporal attention manipulated by cue affected contrast psychometric function by response gain, indicating that multiplying the contrast response of the visual target occurring at the selected point in time by a fixed factor is a crucial way for temporal attention to modulate perceptual processing.

## Introduction

There is a conflict between the numerous visual information flooding our eyes every moment and the limited resources in the human brain that can be used to process them. To solve this incompatibility, a crucial procedure named selective attention is employed to select important visual information and then process it with priority while the rest is ignored. The selection by attention can be based on space, feature, or time termed spatial attention, feature-based attention, and temporal attention respectively. Because of the importance of attention in visual information processing, perception and behavior, its mechanisms have been intensively explored with psychophysical, neuroimaging, and neurophysiological methods (Maunsell and Treue, 2006; Carrasco, 2011; Nobre and van Ede, 2018).

**Figure 1:**
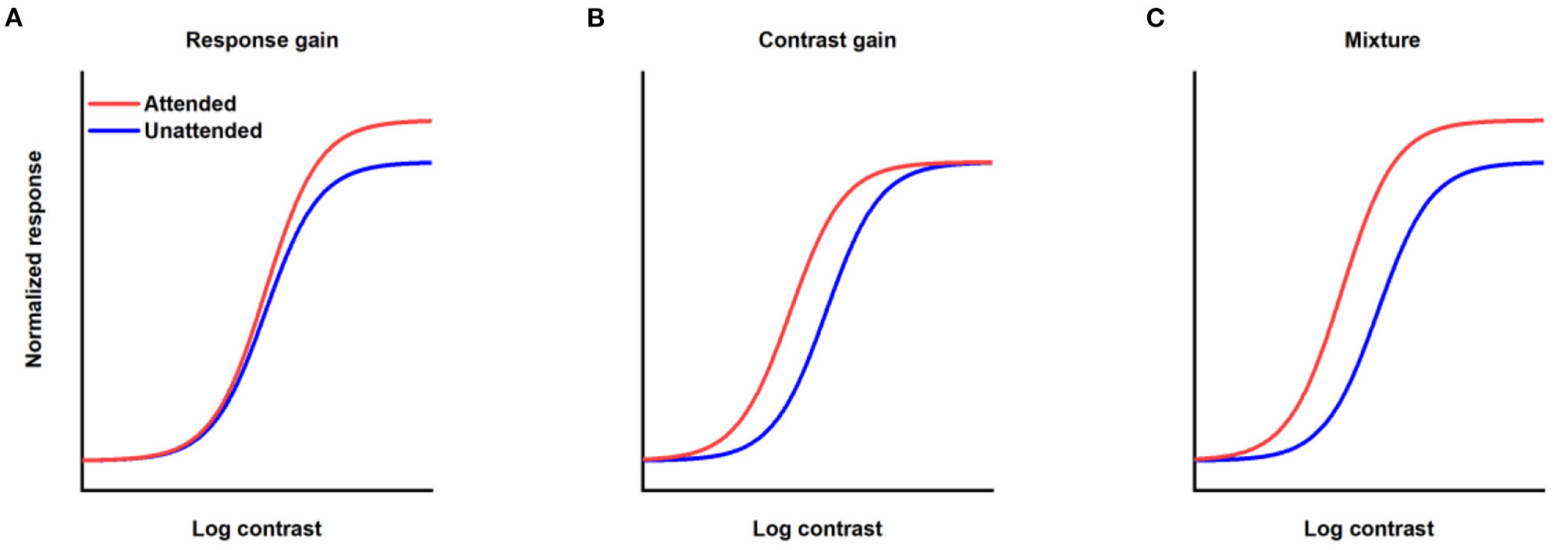
Different forms of the effects of attention modulation on contrast response function. **(A)** Response gain. **(B)** Contrast gain. **(C)** A mixture of response and contrast gains. Red lines represent responses as a function of contrast when stimuli are attended. Blue lines denote responses to unattended stimuli.

How attention affects contrast response function (CRF) is one of the key questions in research on attention. In theory, when the stimulus is selected by attention, the corresponding contrast response function could be modulated by attention with different patterns (Sclar et al., 1989). CRF could be shifted leftwards with obvious alteration in the response to the visual stimulus with intermediate contrast and not others (contrast gain, Figure 1B). Attention could also drive CRF upwards with an enhancement of the response to the stimulus with a fixed scale regardless of the contrast of the stimulus (response gain, Figure 1A). Moreover, attention could mediate CRF in a way just like a mixture of response and contrast gains (Figure 1C). The distinction among discrepant gain patterns of attention modulation is essential, since contrast gain implies that attention acts by increasing the effective contrast of the stimulus, which is equivalent to a reduction in the threshold of the visual system by attention, while response gain reflects that attention changes the input/output transformation of the visual system or how much a specified increase in contrast results in an improvement in response. Empirically, different modulation patterns including response gain (Morrone et al., 2002; Ling and Carrasco, 2006; Pestilli et al., 2007), contrast gain (Reynolds et al., 2000; Martínez-Trujillo and Treue, 2002; Ling and Carrasco, 2006; Li et al., 2008) and mixture gain (Huang and Dobkins, 2005; Williford and Maunsell, 2006; Buracas and Boynton, 2007) have been found in experiments investigating spatial attention and its effects on CRF. Changing the relative size of the attention field to the stimulus from small to large can lead to a transformation in the gain pattern of spatial attention on CRF from response gain to contrast gain (Reynolds and Heeger, 2009; Herrmann et al., 2010; Itthipuripat et al., 2014), demonstrating the flexible modulation of spatial attention. The impact of feature-based attention on CRF is not a simple copy of its spatial counterpart. Some studies support that regardless of the size of the featural extent of the attention field, attention to a visual feature only leads to changes in response gain (Reynolds and Heeger, 2009; Herrmann et al., 2012). Other work has found a pure response gain for feature-based attention with a wide feature focus, but a combination of an increase in the effective sensory input strength and scaling in the responses with a narrow feature focus (Schwedhelm et al., 2016). The dissimilar gain patterns for spatial and feature attention signify the dissimilar characteristics of different types of attention.

In contrast to the plentiful studies concerning the influence of spatial and feature-based attention on CRF, there are few investigations about the effects of temporal attention on CRF. It is widely accepted that temporal attention can modulate visual perception (Morillon and Barbot, 2013). Empirical evidence involves that targets appearing at moments predicted by the rhythmic structure which attract more temporal attention due to the predictability of their occurrence gain perceptual benefits compared with those embedded in an arrhythmic structure whose unforeseeable occurrences lead to less involvement of temporal attention on target processing (Mathewson et al., 2010; Cravo et al., 2013; de Graaf et al., 2013). A perceptual tradeoff has also been revealed for voluntary temporal attention, with the evidence that when attention is directed to a cued point in time, there are perceptual improvements at the anticipated time and also impairments at the ignored time point (Denison et al., 2017). Moreover, it has been demonstrated that temporal attention recruits both stimulus enhancement (amplifying both relevant signal and irrelevant noise) and signal enhancement (exclusively increasing the gain of the target signal) to improve the perception of a target signal (Ramirez et al., 2021). These reported effects of temporal attention on perception only indicate that just like spatial and feature-based attention, temporal attention can lead to changes in CRF, but gives no clue to the detailed pattern by which temporal attention alters CRF. A psychophysical study (Rohenkohl et al., 2012) and its subsequent EEG- recording counterpart (Cravo et al., 2013) from the team of Nobre asked subjects to discriminate the orientations of targets with various contrasts that were embedded in a stream of noise patches separated by a fixed (regular condition) or jittered (irregular condition) intervals and fitted the collected behavior data with psychometric functions for contrast sensitivity for each condition. They found that temporal attention generated from rhythmic structure modulated contrast psychometric function by contrast gain. Additionally, only in the regular but not the irregular condition the EEG study revealed: (1) a strong correlation between the delta pre-stimulus phase and the perceptual contrast gain which was indexed by the threshold obtained from the fitted behavior psychometric function, and (2) a concentration of the delta phase around the phase that corresponded to the best performance, suggesting that phase entrainment of low-frequency oscillations is a neural mechanism that can account for the increase in contrast sensitivity by rhythmic temporal attention. It is well known that temporal attention can be generated from different temporal structures such as associations, hazard rates, rhythms, and sequences, and has distinct functional and neural features according to discrepant structure origins (Coull et al., 2000; Correa and Nobre, 2008; Rohenkohl et al., 2011; Trivino et al., 2011; Breska and Deouell, 2014; Correa et al., 2014; Breska and Ivry, 2018; Amit et al., 2019). However, only temporal attention induced by rhythms has been explored in the aforementioned two pieces of research on temporal attention by the team of Nobre, meaning that how temporal attention induced in other settings influences CRF remains unclear. Additionally, the tasks in these two studies were not rendered difficult enough such that observers' performance asymptotes were at nearly 100% accuracy (ceiling), thus leaving insufficient room for response gain (if any) to manifest itself when the targets were embedded in the rhythmic structure (see their Figure 2a in Rohenkohl et al., 2012, Figure 1b in Cravo et al., 2013). In consideration of these limitations, it is necessary to investigate the gain pattern of temporal attention originating from temporal textures other than rhythm. Among them, temporal attention induced by an informative cue which has a strong temporal association with an incoming target is noteworthy, since similar cueing tasks are also commonly used in research on spatial (Ling and Carrasco, 2006; Herrmann et al., 2010) and feature-based attention (Herrmann et al., 2012). This similarity in paradigms makes it possible and promising to directly compare the corresponding results on spatial, feature-based, and temporal attention, which is not only beneficial for our understanding of temporal attention but also helps to form a comprehensive picture of the underlying mechanisms of attention and whether the mechanisms of different types of attention parallel each other.

In the aim of investigating how cue-induced temporal attention affects CRF, we adopted a temporal-cueing paradigm with audio cues composed of different frequency components to direct attention to distinct time points, combined with the variations in the stimulus contrast to gain psychophysical functions under different attentional states. Computational modeling and fitting were conducted to determine how CRFs were altered by temporal attention.

## Methods

### Observers

Seven subjects (age 20–23 years, 5 males, 2 females) participated in the experiments. All subjects were naïve to the purpose of the experiments. They had normal or corrected-to-normal vision and provided informed consent. The sample size was calculated with the G^*^POWER software (Faul et al., 2007) version 3.1.9.7 for F test (repeated measures ANOVA, within factors), using 0.40 as the effect size of F which was computed from the results described in a study conducted similar experiment protocol to assess whether temporal attention improved performance to the same extent across the visual field (Fernández et al., 2019), an error probability of 0.05, a power of 0.95, correlation among repeated measures of 0.5, and non-sphericity correction of 1. The software which was widely used in behavioral science during experimental planning to appraise the required number of participants (Prete et al., 2022; Trißl and Bäuml, 2022) suggested a sample size of 6, but considering a potential dropout in the process of experiment, 7 participants were finally recruited. The number of subjects in the present study was comparable to those listed in previous studies investigating the effects of attention on CRF (Ling and Carrasco, 2006; Herrmann et al., 2010). According to a study on psychophysical statistics (Anderson and Vingrys, 2001), the significant effect revealed with this sample size exists in the majority of the average population. All experimental procedures conformed to the ethical standards of the Ethical Committee of Sichuan University of Science and Engineering and the guidelines of the Declaration of Helsinki.

### Apparatus

A gamma-corrected 23.8-inch liquid crystal display (LCD) monitor (TITAN ARMY, T24FG, Shenzhen, China, 1,920 × 1,080 pixels, 100 Hz refresh rate) was used to display the visual stimuli with a mean luminance of 15.5 cd/m^2^. The subject was seated 57 cm in front of the screen with his/her head stabilized by a chin rest. The eye movements were monitored by an infrared imaging-based eye tracker (Tobii X60; Tobii Technology AB, Stockholm, Sweden). Stimulus presentation and data collection were achieved using MATLAB (MathWorks) with Psychtoolbox extension (Brainard, 1997; Pelli, 1997). Audios were presented *via* computer speakers.

### Stimuli and procedure

**Figure 2:**
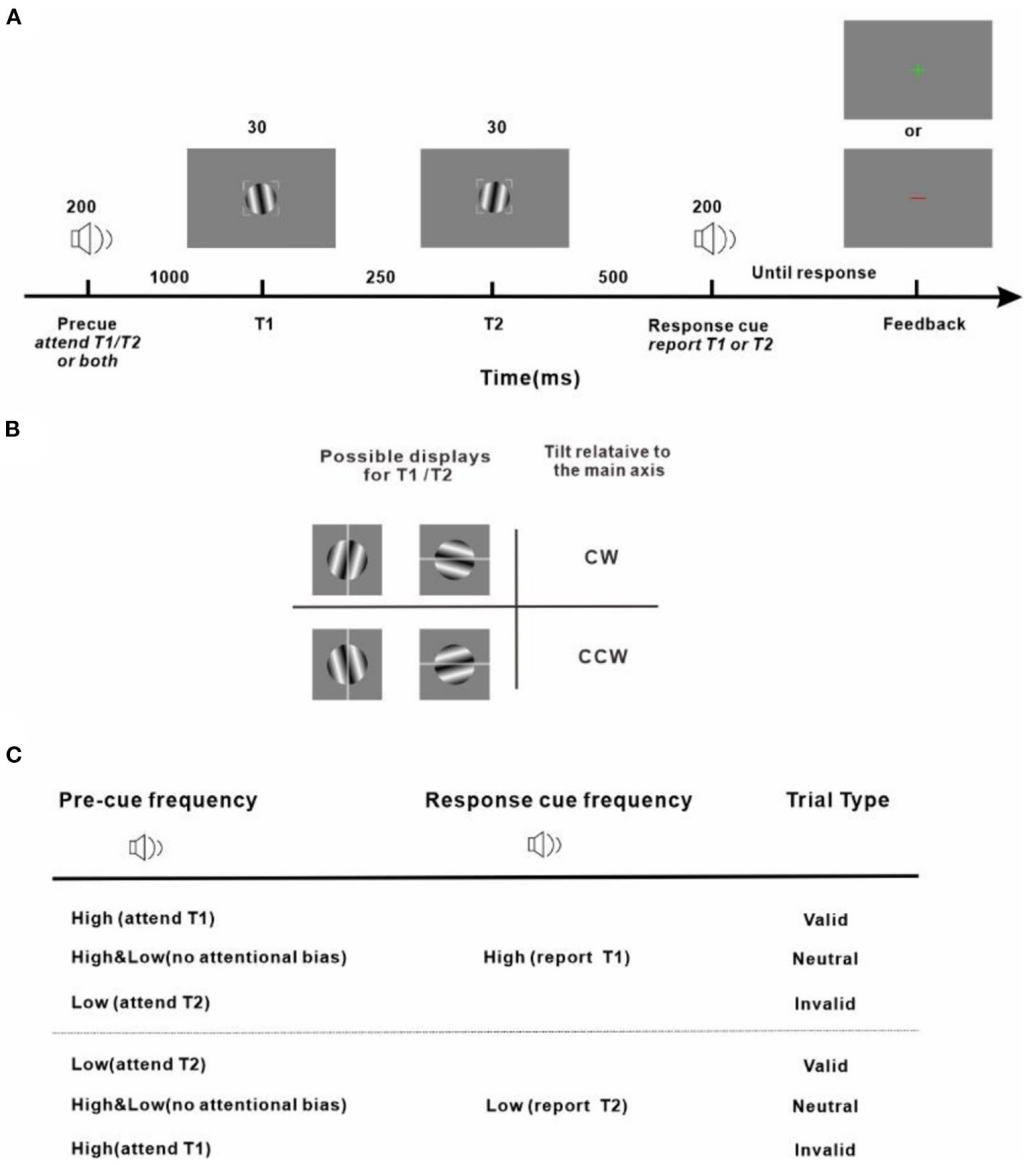
Experiment protocol and response strategy **(A)** Trial sequence. Observers were asked to judge the tilt (clockwise or counter-clockwise) of a sinusoidal grating patch (T1 or T2, indicated by the response cue) from its main axis. **(B)** Stimulus display and response scheme. Observers reported clockwise vs. counter-clockwise tilts relative to either the vertical or horizontal axis depending on which was the closest cardinal axis of the target to be reported (indicated by the gray line for demonstration purposes and not shown during the experiment) by pressing one of two keys on the keyboard. All stimuli, pre-cues, and response cues were presented in randomly interleaved trials. Tilt magnitudes were determined for each observer using a staircase procedure. **(C)** Attention manipulation strategy with different pre-cue and response-cue correspondence. In valid trials, attention was correctly cued to the target to be reported. In invalid trials, attention was misdirected by the pre-cue to the stimulus that was not the reported one at the end of the trial. In neutral trials, there was no attentional preference for the two stimuli in the stream. The protocol is an adapted version of the well-established experimental design to investigate the effect of temporal attention on perception (Denison et al., 2017; Denison and Yuval-Greenberg, 2019; Fernández et al., 2019; Denison and Carrasco, 2021).

Our protocol is an adapted version of the well-established experimental design to investigate the effect of temporal attention on perception (Denison et al., 2017; Denison and Yuval-Greenberg, 2019; Fernández et al., 2019; Denison and Carrasco, 2021). Stimulus placeholders being presented throughout all trials and experimental sessions were used to eliminate spatial uncertainty and assist fixation which were corners of a 2° × 2° gray square outline centered on the center of the screen with a width of 0.08° (Figure 2A). Subjects were instructed to fixate within the square enclosed by the placeholders while performing the task. An audio pre-cue was played for 200 ms to signify the start of a trial. The pre-cue could be high-frequency (4,800 Hz) or low-frequency (600 Hz) pure tone, or their combination. In total of 1,000 ms after the pre-cue, a target (T1) was presented for 30 ms which was located within the placeholders and at the center of the screen. In total of 250 ms after the disappearance of T1, another target (T2), appeared at the center of the screen. T1 and T2 were both Gabor patches (3° × 3°, σ = 0.4°, 4 cycles/deg), tilted slightly away from either horizontal or vertical (randomized on each trial). The tilts of T1 and T2 were the titrated threshold determined in a pretest with a value of 1.39 ± 0.18° (Mean ± Standard Error of Mean, averaged across the subjects). Tilts and axes were independent for T1 and T2. An audio response cue (pure tone of high-frequency with 4,800 Hz or low-frequency with 600 Hz) with 200 ms duration was played 500 ms after the vanishing of T2. Observers were instructed to discriminate the orientation of the target indicated by the response cue (report T1 for high-frequency response cue, report T2 for low-frequency response cue) and indicated whether this target was tilted clockwise (CW) or counter-clockwise (CCW) from its closest cardinal axis by pressing one of two keys on a keyboard (Figure 2B). A visual feedback was sent at the center of the screen (correct: a green cross; incorrect: a red line) after the key response. The next trial began after a random intertrial interval (ITI) between 1,000 to 1,500 ms. Those trials that had reaction times (time interval between the response cue and the response onset, RT) shorter than 150 ms were considered incorrect to prevent guessing. Trials with fixation breaks (the fixation deviated more than 2 degrees from the center of the screen) were stopped immediately without being counted and repeated at the end of the run. Accuracy was emphasized in performing the task. The purpose of adopting two options (vertical and horizontal) for the main axes of Gabor patches was 2 fold: firstly, decreasing the number of trials in which T1 and T2 were the same; secondly, preventing observers from implementing a tactic of judging whether the two consecutive Gabor stimuli were identical or different and using this information to benefit orientation discrimination.

The audio pre-cue was adopted to introduce a preferential allocation of attentional resources toward T1 or T2 or none of them when the subject was performing an orientation discrimination task. Depending on the relationship between the pre-cue and the response cue, the pre-cue was valid if the two were matched, meaning that the pre-cue correctly directed attention to the behavior-related target and was invalid if the two cues were not of the same type, meaning that the pre-cue provided wrong guidance and the behavior-related target was neglected. The pre-cue which was a mixture of the high-frequency and low-frequency tones was neutral and provided no useful information on which of the upcoming targets should be paid attention to. The pre-cues were valid/neutral/invalid in 60/20/20% of the total trials. Before experiments got started, subjects were explicitly told that the pre-cues were informative regarding the targets whose orientation would be discriminated and reported at the end of each trial and that there was a benefit in using the pre-cues to perform the task.

The contrasts of T1 and T2 within a trial were the same but varied from trial to trial. To obtain a complete contrast response function, in each trial the contrast for the two targets was randomly chosen from a set of contrasts ranging from 6 to 67% in 7 log increments.

In a pretest, each observer's orientation discrimination threshold for the tilt of the Gabor patch from its main axis was measured with a three-down one-up staircase procedure to find out the 79%-correct points for the discrimination without differentiating the threshold for each target time (T1 or T2). The threshold value of each subject was used as the tilted angles of Gabor to its main axis in the following formal experiments.

Subjects were asked to complete a number of blocks of 280 trials. For each subject, data collection was stopped when the R^2^ of fit of one of his/her CRFs under valid/neutral/invalid conditions across the reported targets was more than 0.9 to assure the quality of the fitting. Since data noises were not identical among subjects, discrepant numbers of blocks thus different numbers of trials were recorded for each subject. Four subjects completed 10 blocks (2,800 trials). Two subjects completed 12 blocks (3,360 trials). One subject finished 15 blocks (3,920 trials).

### Data analysis and statistics

Data analyses were conducted with the Statistical Package for Social Sciences (SPSS, Inc.) and OriginPro software (OriginLab Corporation).

For each subject, perceptual sensitivity values (*d'*) and reaction times were assessed for each pre-cueing condition (valid, invalid, and neutral) and each contrast level. Sensitivity was calculated according to the formula:


$$\begin{array}{l} d^{\prime }=z\left[hit\;rate\right]-z\left[false\;alarm\;rate\right] \end{array}$$

where *z* corresponds to the inverse normal (*z* score). A correct response to the tilt of the stimulus relative to its closest cardinal axis was regarded as a hit while a wrong reply was considered as a false alarm.

The fitting procedure was similar to previous studies to investigate the influence of spatial (Herrmann et al., 2010) and feature-based attention (Herrmann et al., 2012) on CRF. In detail, for each pre-cueing condition and each contrast level, the mean sensitivity value across subjects was calculated. The averaged *d'* values were fitted (*via* non-linear least-squares) to the Naka–Rushton contrast response model (Albrecht and Hamilton, 1982; Sclar et al., 1990):


$$\begin{array}{l} d^{\prime }\left(c\right)\;=\;\frac{d_{\max}*c^{n}}{c^{n}+c_{50}^{n}}+M \end{array}$$

where *d'*(c) represents sensitivity *d'* as a function of contrast *c, c*_50_ is the contrast corresponding to half the saturating response (threshold), *n* is the exponent which determines the slope of the function, *d*_*max*_ controls the asymptote performance at high contrasts, and M represents the response at the lowest contrast level which is 0 for *d'*. *c*_50_ and *d*_*max*_ are free parameters varied for different pre-cueing conditions (valid, invalid, and neutral), while the exponent *n* was treated as one free parameter, constrained to have the same value across conditions.

**Figure 3:**
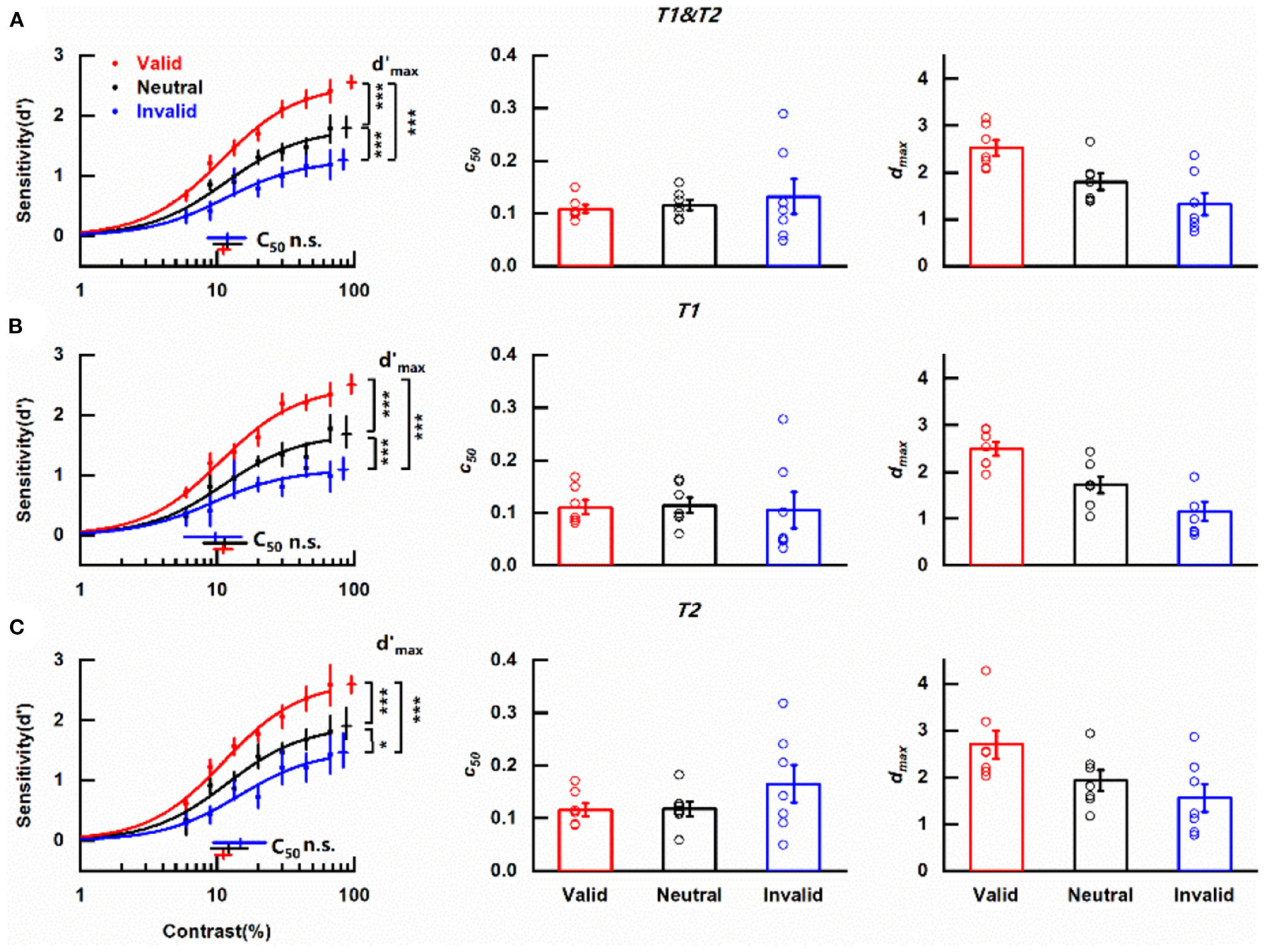
Effects of cue-induced temporal attention on contrast sensitivity **(A)** Left column: contrast response functions for different pre-cueing conditions. Data were collapsed across the reported targets. Each data point denotes the mean across observers under the corresponding pre-cueing condition. Red, blue, and black symbols and lines indicate valid, invalid, and neutral pre-cues respectively. Error bars on data points represent within-subject standard errors of the mean, after the removal of the between-subject variability (Cousineau, 2005; Morey, 2008; Franz and Loftus, 2012; Baguley, 2013). Error bars on parameter estimates are 90% confidence intervals, obtained by bootstrapping. Middle column: parameter estimates of threshold *c*_50_ for trials with different pre-cues for each participant. Open symbols denote parameter estimates of fitting curves of individual subjects. The bars indicate the means of the parameter estimates across the subjects under corresponding conditions. Error bars represent standard errors of the mean. Right column: same as the middle column but for parameter estimates of asymptote performance *d*_*max*_. ^*^p < 0.05, ^***^p < 0.001. **(B)** Same as **(A)**, but only including the trials in which the reported target was T1. **(C)** Same as **(A)**, but only including the trials in which the reported target was T2.

The confidence intervals of the fitted *d*_*max*_ and contrast *c*_50_ were determined by a bootstrap procedure (Figure 3A). In detail, a resampled dataset was generated by randomly resampling with replacement of individual psychophysical trials, which was refitted subsequently. We repeated this procedure involving resampling and refitting 1000 times to generate bootstrap distributions of the fitted parameters from which the confidence intervals for each parameter were extracted. Another bootstrap procedure (Yuval-Greenberg et al., 2014; Grubb et al., 2015) was used to determine whether there were significant changes in two key parameters (*d*_*max*_, *c*_50_) between two discrepant pre-cueing conditions (such as trials with valid vs. invalid pre-cues). Specifically, we randomly shuffled the labels of these two conditions to be explored, separately for each contrast level, and separately for each subject. Based on the new labels, the shuffled data was refitted to yield new parameter estimates for *d*_*max*_and *c*_50_ for each pre-cueing condition respectively, followed by the calculation of the difference in *d*_*max*_ and *c*_50_between these two conditions. After 1,000 times repetitions of this procedure, a null distribution for the difference of *d*_*max*_ between trials with valid and invalid pre-cues was generated. The difference in *d*_*max*_ observed in our actual experiment was then compared with the null distribution. The *P-*value reported is the proportion of null distribution values greater than or equal to the actual change in *d*_*max*_to reflect the probability of response gain change. *P*-value for the difference in *c*_50_ was computed in the same manner except that the *P*-value reported is the proportion of null distribution values less than or equal to the actual change in *c*_50_to illustrate the likelihood of contrast gain change.

For each observer, the psychometric functions were also fitted for different pre-cueing conditions. The parameter estimates of the fitting were recorded and analyzed by one-way ANOVA for repeated measures to evaluate the influence of temporal attention induced by cue by including pre-cue type as the factor (valid, invalid, and neutral).

## Results

### Cue-induced temporal attention modulates contrast perception by response gain

Considering the main purpose of assessing the modulation of temporal attention induced by the pre-cue, data were collapsed across the reported targets. The mean contrast response functions under discrepant pre-cueing conditions (valid, invalid, and neutral) were shown in Figure 3A. It could be seen that no matter what the pre-cue was, *d'* increased with the increase of target contrast and saturated at high contrast, forming a typical s-shape curve. Importantly, the two psychometric functions representing perceptual sensitivity under valid and invalid pre-cueing conditions were not overlapped with each other but had an order with the one obtained in trials with valid pre-cues being always above the line indicating invalid pre-cueing condition. The curve depicting the neutral condition constantly laid between the other two psychometric functions. The clear separation of these three functions demonstrates the influence of temporal attention on the perception of contrast.

A decrease in threshold *c*_50_ with no change in asymptote *d*_*max*_ induced by attention is typical in the contrast gain model while the response gain model is characterized by an attentional boost in asymptote *d*_*max*_ in concomitant with no alternation in *c*_50_. The parameters of the fitted psychometric functions representing trials that pre-cues provided valid/neutral/invalid indication of the target to be reported were compared to determine how cue-induced temporal attention affects perceptual sensitivity. *c*_50_ values for valid, neutral and invalid pre-cues were 0.111 (90% confidence interval = [0.101, 0.124]), 0.118 (90% confidence interval = [0.094, 0.151]), and 0.119 (90% confidence interval = [0.085, 0.163]) respectively (Figure 3A, left column, Supplementary Figure 1B) and were not significantly different from each other (valid vs. neutral, *p* = 0.314, Supplementary Figure 2B; valid vs. invalid, *p* = 0.301, Supplementary Figure 2D; neutral vs. invalid, *p* = 0.501, Supplementary Figure 2F). The scenario was different for *d*_*max*_ (Figure 3A, left column). The *d*_*max*_ value of CRF representing valid pre-cue was 2.55 (90% confidence interval = [2.45, 2.66], Supplementary Figure 1A), which was not only significantly larger (*p* < 0.001, Supplementary Figure 2C) than its counterpart (1.26, 90% confidence interval = [1.10, 1.44], Supplementary Figure 1A) obtained under invalid pre-cueing condition but also differed significantly (*p* < 0.001, Supplementary Figure 2A) from the *d*_*max*_ value of the CRF under neutral pre-cueing condition (1.79, 90% confidence interval = [1.64, 1.99], Supplementary Figure 1A). Additionally, there was a significant difference (*p* < 0.001, Supplementary Figure 2E) between the *d*_*max*_ values of the two CRFs under neutral and invalid pre-cueing conditions. A parallel pattern was revealed with the analysis of the psychometric functions from individual observers. Pre-cue type did not show a significant influence on *c*_50_ values [*F*_(1, 6)_ = 0.420, *p* = 0.541, η^2^ = 0.065; Figure 3A middle column] while *d*_*max*_ values were affected significantly by pre-cue type [*F*_(1, 6)_ = 12.059, *p* = 0.013, η^2^ = 0.668; Figure 3A right column]. These observed effects of attention modulation on *d*_*max*_ in combination with the fact that no influence of attention was found on *c*_50_ from both the average and the individual data indicate that temporal attention induced by cue modulates perceptual sensitivity *d' via* response gain. Furthermore, the revealed differences in perceptual sensitivitie*s* between the valid and neutral pre-cueing conditions illustrate the enhancement of temporal attention on the perception of the attended target (attentional benefit) while the discovered decrease in perceptual sensitivity when the temporal cue was invalid compared with the neutral pre-cueing condition demonstrates the impairment of temporal attention on the perception of ignored distractors (attentional cost).

A previous study using a similar paradigm to investigate the effect of temporal attention on sensitivity has demonstrated that sensitivity *d'* was comparable for T1 and T2 (Denison et al., 2017). We also divided our data based on whether the reported target was T1 or T2 and analyzed the CRFs of the two subgroups for each pre-cueing condition to assess whether temporal attention modulated the perception of T1 and T2 with the same pattern. Regardless of the reported target, the CRF denoting perceptual sensitivity under valid pre-cueing condition was always at the top, with the psychometric function representing neutral pre-cueing condition in the middle and the curve describing invalid pre-cueing condition at the bottom of the three (Figure 3B left column, Figure 3C left column). When the reported target was T1, the *c*_50_ values for different pre-cueing conditions were quite similar (valid: 0.111, 90% confidence interval = [0.095, 0.129]; neutral: 0.114, 90% confidence interval = [0.079, 0.164]; invalid: 0.097, 90% confidence interval = [0.057, 0.151]) (Figure 3B, left column) and did not differ significantly from each other (valid vs. neutral, *p* = 0.897; valid vs. invalid, *p* = 0.466; neutral vs. invalid, *p* = 0.667). However, CRFs representing discrepant pre-cueing conditions had significantly different *d*_*max*_ values (valid vs. neutral, *p* < 0.001; valid vs. invalid, *p* < 0.001; neutral vs. invalid, *p* < 0.001) with the largest *d*_*max*_ value under the valid pre-cueing condition (2.50, 90% confidence interval = [2.35, 2.67]), the smallest *d*_*max*_ value under invalid pre-cueing condition (1.09, 90% confidence interval = [0.93, 1.30]) and the medium *d*_*max*_ value when the pre-cue was neutral (1.68, 90% confidence interval = [1.47, 1.98]). The analysis of one-way ANOVA for repeated measures showed that pre-cue type did not significantly impact *c*_50_ value [*F*_(1, 6)_ = 0.03, *p* = 0.868, η^2^ = 0.005; Figure 3B middle column] but had a significant influence on *d*_*max*_ value [*F*_(1, 6)_ = 23.40, *p* = 0.003, η^2^ = 0.796; Figure 3B right column]. The same pattern was found when T2 was the reported target. There were no significant differences (valid vs. neutral, *p* = 0.509; valid vs. invalid, *p* = 0.075; neutral vs. invalid, *p* = 0.435) among the similar *c*_50_ values of different pre-cueing conditions (valid: 0.112, 90% confidence interval = [0.096, 0.126]; neutral: 0.122, 90% confidence interval = [0.091, 0.167]; invalid: 0.147, 90% confidence interval = [0.094, 0.228]) (Figure 3C, left column) but the *d*_*max*_ values for discrepant pre-cueing conditions (valid: 2.59, 90% confidence interval = [2.45, 2.74]; neutral: 1.90, 90% confidence interval = [1.69, 2.20]; invalid: 1.46, 90% confidence interval = [1.21, 1.79]) differed significantly from each other (valid vs. neutral, *p* < 0.001; valid vs. invalid, *p* < 0.001; neutral vs. invalid, *p* = 0.014). Meanwhile, the significant impact of pre-cue type on *d*_*max*_ was observed with one-way ANOVA for repeated measures [*F*_(1, 6)_ = 4.14, *p* = 0.043, η^2^ = 0.668; Figure 3C right column] but pre-cueing method was not a significant influential factor for *c*_50_ [*F*_(1, 6)_ = 1.13, *p* = 0.330, η^2^ = 0.158; Figure 3C middle column]. To directly appraise whether there were distinct patterns of attention modulation on CRFs for different reported targets, a two-way ANOVA for repeated measures including pre-cue type (valid, neutral, and invalid) and reported target (T1, T2) as factors was used to analyze the parameter estimates of CRFs fitted from data of individual observer. For *c*_50_, there was neither a significant main effect of reported target [*F*_(1, 6)_ = 4.81, *p* = 0.071, η^2^ = 0.445] nor pre-cue type [*F*_(2, 12)_ = 0.28, *p* = 0.642, η^2^ = 0.044]. The interaction effect of these two factors was also insignificant [*F*_(2, 12)_ = 4.23, *p* = 0.076, η^2^ = 0.413]. For *d*_max_, the main effect of reported target was significant [*F*_(1, 6)_ = 7.38, *p* = 0.035, η^2^ = 0.551], reflecting a significantly higher *d*_max_ for late T2 than early T1. The main effect of pre-cue type was also significant [*F*_(2, 12)_ = 10.18, *p* = 0.013, η^2^ = 0.629]. There was no significant interaction effect of these two factors [*F*_(2, 12)_ = 0.21, *p* = 0.802, η^2^ = 0.034]. Based on these results, it could be concluded that temporal attention which was induced by cue modulated psychometric function by response gain no matter whether the reported target was T1 or T2, indicating the independence of the modulation pattern on the order of the reported target.

We also evaluated whether the reported target was near horizontal or vertical would affect the gain pattern of temporal attention induced by cue on CRF (see Supplementary material for details and results). The sensitivity *d*' for targets with vertical main axis was significantly larger (*p* < 0.001) than the sensitivity *d*' for near-horizontal targets indicating that visual perception varies with stimulus orientation. But the reaction speed for the targets with discrepant main axes did not differ significantly (*p* = 0.113). Importantly, whatever the orientation of the main axis of the reported target, the *c*_50_values of CRFs denoting valid/neutral/invalid conditions did not have significant differences (all *ps* ≥ 0.621). Meanwhile the *d*_*max*_ values of these CRFs differed significantly (all *ps* ≤ 0.005). The results illustrated cue-induced temporal attention modulated CRF *via* response gain regardless of the orientation of the reported target.

Moreover, based on the assessment, the best-fitting value for the exponent n of the psychometric functions was 1.55 for our data (Supplementary Figure 3). In previous research fitting CRF with Naka–Rushton contrast response model, different n values were obtained. In the study of Sclar et al., the *n* value for different types of neurons ranged from 1.2 to 3.0 (Sclar et al., 1990). In the study of Herrmann et al., the values of the exponent n were 2.48 and 2 for exogenous and endogenous spatial attention respectively (Herrmann et al., 2010). We also used these reported exponent values in previous studies to evaluate the influence of the n value, and got the same conclusion (data not shown).

Together, these results illustrate that in our experiment perceptual sensitivity for contrast was modulated by temporal attention which was manipulated by cue. Additionally, our data were fitted by the response gain model but not the contrast gain model or the mixture model. Meanwhile, our data also showed perceptual tradeoffs due to temporal attention, illustrated by the enhanced perceptual sensitivity for the target occurring at the attended time point and the worsened perception of the distractor happening at the unattended time point.

### Impacts of cue-induced temporal attention on reaction time

**Figure 4:**
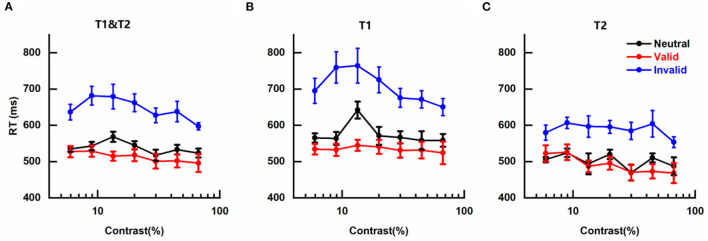
Effects of cue-induced temporal attention on reaction time **(A)** mean reaction times (RTs) across targets, plotted for different pre-cues as a function of contrast intensity. Error bars on data points denote within-subject standard errors of the mean, after the removal of the between-subject variability (Cousineau, 2005; Morey, 2008; Franz and Loftus, 2012; Baguley, 2013). **(B)** same as **(A)** but only for trials in which the reported target was T1. **(C)** same as **(A)** but only for trials in which T2 was the reported target.

The effects of cue-induced temporal attention on RT were also observed with a similar pattern as for perceptual sensitivity *d'* (Figure 4), illustrated by fastest RTs on trials with valid pre-cues (Mean ± SEM: 0.511 ± 0.015 s), slowest RTs on trials that were invalid pre-cued (0.645 ± 0.019 s), and intermediate RTs on neutral trials (0.538 ± 0.008 s).

The mean RTs of different conditions were submitted to a three-way analysis of variance (ANOVA) for repeated measures with pre-cue type (valid, invalid, neutral), target contrast (seven levels), and reported target (T1, T2) as three factors. It was not surprising that a significant main effect of pre-cue type was observed [*F*_(2, 12)_ = 15.696, *p* < 0.001, η^2^ = 0.723]. There was also a significant main effect of reported target [*F*_(1, 6)_ = 55.438, *p* < 0.001, η^2^ = 0.902], reflecting faster discrimination for the tilt of T2 than T1 (Figures 4B, C), which might be due to the subject being more prepared for orientation discrimination at the appearance of late T2 than early T1. No significant main effect of target contrast was found [*F*_(6, 36)_ = 3.318; *p* = 0.053, η^2^ = 0.356], indicating that in our experiment RT was not influenced by the contrast of target, and other factors that could lead to changes in RT such as motor preparation (Correa, 2012) or criterion changes (Carrasco and McElree, 2001) played more influential roles on RT. The two-way interaction effects of the three pairs were not significant [Pre-cue type × Target contrast: *F*_(12, 72)_ = 1.299, *p* = 0.303, η^2^ = 0.178; Pre-cue type × Reported target: *F*_(2, 12)_ = 3.975, *p* = 0.064, η^2^ = 0.398; and Target contrast × Reported target: *F*_(6, 36)_ = 2.554, *p* = 0.093, η^2^ = 0.299]. The three-way interaction effect of all three factors was also not significant [*F*_(12, 72)_ = 1.297, *p* = 0.307, η^2^ = 0.178].

We also submitted the mean RTs of different conditions to a three-way analysis of variance (ANOVA) for repeated measures with pre-cue type (valid, invalid, and neutral), target contrast (seven levels), and the main axis of the reported target (horizontal, vertical) as three factors to gauge the influence of the main axis of the reported target on RT (see Supplementary material for details and results). The results did not show the main effect of the main axis of reported target or any other significant two-way interaction effects involving this factor.

The results on RT demonstrate that the observed changes in contrast sensitivity induced by temporal attention could not be attributed to the speed-accuracy tradeoff.

## Discussion

In the present study, we demonstrated that directing attention to a pre-cued time point affects the perception of relevant events, with enhanced contrast sensitivity for the target occurring at the attended time (attentional benefit), and deteriorated contrast sensitivity for the distractor happening at the unattended time (attentional cost). Importantly, we compared the psychometric functions for contrast sensitivity when attention was directed to different time points by temporal cue, and found that this cue-induced temporal attention resulted in a multiplicative magnification in the psychometric function, a typical characteristic of response gain model of attention, indicating that temporal attention can modulate visual perception by proportional scaling of the response to contrast.

There are some inconstancies in the definitions and taxonomies in research on attention. Selective attention is a broad construct that contains the set of brain functions that prioritize and select relevant information to guide consequent behavior in the present study. Many factors can lead to such preference in processing certain information, among which task goal and expectation based on the probability of items are two noticeable modulatory signals. The preferences induced by goal and expectation are conceptually dissociable, but they often work together to prioritize probable task-relevant events for neural processing and/or to filter irrelevant events (Nobre and Kastner, 2014). Hence, we do not strictly distinguish attention induced by goal and expectation in the present study. This is different from a suggested strict dichotomy in which attention and expectation are two distinct constructs. The former refers only to task relevance or task goal and the latter corresponds to prior likelihood.

The ability of temporal attention to affect visual perception is well recognized. A typical illustration is the widely observed high and low perceptual sensitivities for the events occurring at the anticipated and the unexpected time respectively ascribed to the modulation of temporal attention (Correa et al., 2005; Rohenkohl et al., 2012). Our study adds new empirical evidence for the power of temporal attention to modulate perception which includes the boost for the attended target and the suppression of the disregarded distractor (Denison et al., 2017; Denison and Yuval-Greenberg, 2019; Denison and Carrasco, 2021). In addition, our work goes further than previous research and extends to a follow-up question: how does temporal attention exert its effect on visual perception? Our results show that temporal attention induced by cue affects contrast psychometric function by response gain, indicating that temporal attention multiplicatively amplifies the responses to stimuli as a function of contrast intensity which consequently leads to changes in perceptual processing for visual stimuli. However, Rohenkohl et al. have observed the typical phenomenon of contrast gain in their behavioral study on temporal attention-a leftward shift of the contrast psychometric function induced by temporal attention, illustrating that temporal attention enhances the effective contrast of attended target to modulate perception (Rohenkohl et al., 2012). Two possible explanations could be proposed for this inconsistency. The first one could be the ceiling effect. In the work of Rohenkohl et al., the performance accuracies of subjects almost reached ceiling level (100%) when the target had high contrast intensity, leaving no space for response gain to present itself. This possible ceiling effect makes their observed contrast gain change in contrast response function by temporal attention debatable. In our study, the performance accuracies were just around 90% even when the contrast intensity was at its maximal level and the pre-cue was valid, far from the 100% ceiling. The second explanation could be due to the different ways of experimental manipulation in these two studies and the distinction between expectation and goal-directed attention. Both selectively attending to sensory inputs relevant to task goal and expectations based on learned signal probability in incoming sensory signals can modulate neural signals and perception. Wyart et al. (2012) asked subjects to probe the presence of a target, with manipulation of expectation at the block level using a pre-cue at the beginning of each block indicating the prior probability of target occurrence in each of the two predetermined locations and management of the goal-directed attention on a trial level with a pre-cue in each trial indicating the most likely position of the target in that trial. They found that expectation and goal-directed attention had dissociable and disparate impacts on visual contrast sensitivity: expectation increased both the hit rate and false alarm rate of the target detection while goal-directed attention boosted the hit rate but reduced the false alarm rate (see their Figure 1), suggesting that expectation and goal-directed attention do not share the same underlying mechanism. In our research, we adopted a well-established protocol (Denison et al., 2017; Denison and Yuval-Greenberg, 2019; Fernández et al., 2019; Denison and Carrasco, 2021) using pre-cue in each trial indicating the goal-relevant target (T1 or T2) in that trial to manipulate goal-directed attention at a trial level. Additionally, in our experiment, the reported target had the equal possibility to be T1 or T2, to be near horizontal or near vertical, to tilt clockwise or counterclockwise relative to its closest cardinal axis, and the visual stimuli in our protocol were predictably timed, indicating that no meaningful information on the prior probability of the reported target could be used to raise expectation and thus affected subsequence perception and behavior. Given all these facts, it is reasonable to claim that expectation did not play important role in our results but only task goal or relevance took effect. On the contrary, in the work of Rohenkohl et al., they asked the subject to judge the orientation of brief targets embedded within temporally regular or irregular streams of noise patches, which means the prior probabilities of the targets were different between these two conditions leading to distinct levels of expectation, hence their results of comparing the contrast sensitivities between the regular and irregular conditions reflected the modulation effects of expectation. The discrepant experiment manipulations in these two studies which led to expectation and goal-directed attention respectively could explain why Rohenkohl et al. modeled their results as contrast gain but response gain was observed in our research. It also raises the possibility that expectation and goal-directed attention modulate CRFs by different gain patterns which is a subject for further research.

The effect of modulation of temporal attention on contrast psychometric function could be impacted by many factors. Studies on spatial attention have demonstrated that the stimulus size and the relative size of the spatial scope of attention can determine whether spatial attention modulates CRF by contrast or response gain (Herrmann et al., 2010; Itthipuripat et al., 2014). A study on feature-based attention has found that, unlike spatial attention, feature-based attention leads to changes in response gain regardless of the size of the featural extent of the attention field (Herrmann et al., 2012). In light of these previous studies, it is natural to ask whether changing the size of the temporal extent of the attention field can lead to changes in the pattern of gain effects in psychometric functions. The present study does not provide a direct answer to this question, even though in our experiment, subjects needed to attend to both of the two stimuli (T1 and T2) occurring at different times when the pre-cue was neutral but only focused on one event (T1 or T2) in trials with invalid or valid pre-cues, meaning that the temporal extent of the attention field under neutral condition was broader than under other conditions. It puts forwards a subject for future studies to systemically vary the size of the temporal extent of the attention field and explore the changes in the attention gain effects in psychometric functions. Besides the size of the temporal extent of the attention field, the type of temporal structure is another possible influential factor worthy of further investigation. As above-mentioned, temporal attention can be generated by different temporal structures such as associations, hazard rates, rhythms, and sequences. These different origins lead to distinct underlying mechanisms for subdivisions of temporal attention. Rigorous experiments are needed to examine whether these subtypes of temporal attention affect psychometric functions with the same pattern.

Divisive normalizations seem to be prevalent in sensory as well as in perceptual and cognitive processing (Heeger, 1992; Carandini and Heeger, 2011). Several studies modeled the effects of attention modulation on CRFs by associating sensory normalization with attentional modulation (Boynton, 2009; Lee and Maunsell, 2009; Reynolds and Heeger, 2009; Beuth and Hamker, 2015; Schwedhelm et al., 2016). Attentional modulation on the gain of the response is posited in these models which alters the contrast sensitivity of neural responses and leads to subsequent changes in CRFs. Among these models, the one developed by Reynolds and Heeger additionally proposed that attention modulates neural activity before normalization (Reynolds and Heeger, 2009). By varying the size of the stimulus conditions and the spread of the attention field, this model exhibits a wide variety of gain patterns by which attention affects CRFs and can account for a wide range of ostensibly conflicting electrophysiological and psychophysical findings (Reynolds and Heeger, 2009). This model predicts that when the scope of spatial attention is large and the stimulus is relatively small, spatial attention modulates the contrast psychometric function by contrast gain, while modulation of spatial attention leads to change in CRF by response gain when attention field is small combined with a comparatively large stimulus. The predictions of the normalized model on how the size of attention field affects the pattern of the mediation of spatial attention on CRF have been verified by consequent psychophysical (Herrmann et al., 2010) and electrophysiological studies (Itthipuripat et al., 2014). Besides spatial attention, the normalized model has also predicted that feature-based attention can only lead to changes in response gain regardless of the size of the featural extent of the attention field which has been also confirmed by a following empirical study (Herrmann et al., 2012). Given its exceptional ability to explain a large body of empirical data and the empirical evidence for its predictions, the normalized model proposed by Reynolds and Heeger becomes a widely accepted model of attention modulation. However, this leading model concerning spatial and feature-based attention is static and cannot account for the time courses of dynamic sensory processes such as temporal attention. In a recent study, Denison et al. generalized the Reynolds and Heeger normalization model of attention to the time domain and proposed a normalization model of dynamic attention which reproduced an empirically observed phenomenon-the perceptual tradeoff caused by temporal attention (Denison et al., 2017; Denison and Carrasco, 2021). Yet it hasn't been known whether the normalization model of dynamic attention proposed by Denison and Carrasco (2021) can produce the response gain change in CRFs by temporal attention as we observed in the present study. Considering that previous attention models involving normalization successfully capture the patterns of attentional modulation on CRFs for spatial and feature-based attention, it is promising to model the effects of temporal attention on CRFs with a normalization model of attention.

Other studies have investigated the modulation patterns of cue-induced spatial (Herrmann et al., 2010) and feature-based attention (Herrmann et al., 2012) on CRFs and find that these types of attention can alter the contrast psychometric function by response gain. Combining our study with these previous works, it can be seen that enhancing the contrast response of stimulus multiplicatively is a general mechanism for cue-induced attention to modulate contrast perception. Moreover, the amplitude of the modulation of temporal attention on contrast sensitivity observed in our study is comparable to the strength of the effect of spatial attention on CRF revealed in the research of Herrmann and coworkers (Herrmann et al., 2010), which demonstrates that non-spatial attention can modulate perception with the same power as the spatial attention.

In conclusion, our results provide empirical evidence that temporal attention manipulated by cue affects contrast psychometric function by response gain, indicating that temporal attention can multiplicatively boost the contrast response of stimuli appearing at the attended time to shape visual perception.

## Data availability statement

The raw data supporting the conclusions of this article will be made available by the authors, without undue reservation.

## Ethics statement

The studies involving human participants were reviewed and approved by the Ethical Committee of Sichuan University of Science and Engineering. The patients/participants provided their written informed consent to participate in this study.

## Author contributions

DH devised the project and drafted the manuscript. CJ, HJ, and ZW collected the data and carried out the data analyses. WL and YC participated in the design of the study and helped write the paper. All authors contributed to the article and approved the submitted version.
