# Supplementary material for: Temporal attention affects contrast response function by response gain
Source: Front Hum Neurosci. 2023 Jan 25;16:1020260. doi: 10.3389/fnhum.2022.1020260 (PMC9905113; doi:10.3389/fnhum.2022.1020260)
Supplement: Supplementary file 1 [file Data_Sheet_1.docx]

**Impacts of Main Axis of Target on Contrast Perception and Reaction Time**

Human visual acuity varies with stimulus orientation, being highest for the orientations of relevant and common visual stimuli in the environment (Annis and Frost, 1973). Orientation anisotropies might play a role in our experiments since the main axes of the Gabor patches could be either vertical or horizontal. Our data were separated based on whether the reported target was near horizontal or vertical. We found evidence for orientation anisotropies. On average, the sensitivity d' for targets with vertical main axis was 1.418 ± 0.036 (mean ± Standard Error of Mean), significantly larger (*p* < 0.001) than the sensitivity d' for near-horizontal targets which was 1.232 ± 0.094. Consequently, we examined the CRFs of the two subgroups for each pre-cueing condition. Regardless of the orientation of the reported target, the CRF denoting perceptual sensitivity under valid pre-cueing condition was always at the top, with the psychometric function representing neutral pre-cueing condition in the middle and the curve describing invalid pre-cueing condition at the bottom of the three (**Supplementary Fig. 4A**, left column, **Supplementary Fig. 4B** left column). When the main axes of reported targets were vertical, CRFs denoting different pre-cueing conditions had rather close *c_50_* values (valid: 0.095, 90% confidence interval = [0.081, 0.109]; neutral: 0.101, 90% confidence interval = [0.073, 0.138]); invalid: 0.101, 90% confidence interval = [0.065, 0.153]) (**Supplementary Fig. 4A**, left column) which did not differ significantly from each other (valid vs. neutral, *p* = 0.657; valid vs. invalid, *p* = 0.721; neutral vs. invalid, *p* = 0.997). However, CRFs representing discrepant pre-cueing conditions had significantly different *d_max_* values (valid vs. neutral, *p* < 0.001; valid vs. invalid, *p* < 0.001; neutral vs. invalid, *p* = 0.005) with the largest *d_max_* value under the valid pre-cueing condition (2.59, 90% confidence interval = [2.45, 2.75]), the smallest *d_max_* value under invalid pre-cueing condition (1.26, 90% confidence interval = [1.08, 1.50]) and the medium *d_max_* value when the pre-cue was neutral (1.78, 90% confidence interval = [1.59, 2.02]). The analysis of one-way ANOVA for repeated measures showed that pre-cue type did not significantly impact *c_50_* value (*F*(1,6)= 1.24, *p* = 0.308, *η^2^* = 0.171; **Supplementary Fig. 4A** middle column) but had a significant influence on *d_max_* value (*F*(1,6)= 10.90, *p* = 0.016, *η^2^* = 0.645; **Supplementary Fig. 4A** right column). The same pattern was found when the reported target was near horizontal. There were no significant differences (valid vs. neutral, *p* = 0.731; valid vs. invalid, *p* = 0.621; neutral vs. invalid, *p* = 0.922) among the similar *c_50_* values of different pre-cueing conditions (valid: 0.134, 90% confidence interval = [0.114, 0.156]; neutral: 0.141, 90% confidence interval = [0.099, 0.199]); invalid: 0.144, 90% confidence interval = [0.085, 0.235]) (**Supplementary Fig. 4B**, left column) but the *d_max_* values for discrepant pre-cueing conditions (valid: 2.54, 90% confidence interval = [2.38, 2.73]; neutral: 1.82, 90% confidence interval = [1.59, 2.15]); invalid: 1.27, 90% confidence interval = [1.05, 1.60]) differed significantly from each other (valid vs. neutral, *p* < 0.001; valid vs. invalid, *p* < 0.001; neutral vs. invalid, *p* = 0.004) . Meanwhile, the significant impact of pre-cue type on *d_max_* was observed with one-way ANOVA for repeated measures (*F*(1,6)= 6.34, *p* = 0.045, *η^2^* = 0.521; **Supplementary Fig. 4B** right column) but pre-cueing method was not a significant influential factor for *c_50_* (*F*(1,6)= 0.45, *p* = 0.572, *η^2^* = 0.070; **Supplementary Fig. 4B** middle column). Based on these results, it could be concluded that temporal attention induced by cue modulated psychometric function by response gain no matter whether the reported target was near horizontal or vertical, indicating the independence of the modulation pattern on the orientation of the reported target.

A three-way analysis of variance (ANOVA) for repeated measures with pre-cue type (valid, invalid, neutral), target contrast (seven levels) and main axis of reported target (vertical, horizontal) as three factors was performed to analyze the mean RTs of different conditions. It was not surprising that a significant main effect of pre-cue type was observed (*F*(2,12) = 15.993, *p* < 0.001, *η^2^* = 0.727). There was no significant main effect of main axis (*F*(1,6) = 3.431, *p* = 0.113, *η^2^* = 0.364), reflecting similar reaction speed for reported targets near vertical and horizontal (**Supplementary Fig. 5 A, B**). A significant main effect of target contrast was found (*F*(6,36) = 3.550, *p* = 0.048, *η^2^* = 0.372) with a P value close to the critical value of 0.05. The two-way interaction effects of the three pairs were not significant (Pre-cue type × Target contrast: *F*(12,72) = 1.670, *p* = 0.206, *η^2^* = 0.218; Pre-cue type × Main axis of reported target: *F*(2,12) = 1.582, *p* = 0.248, *η^2^* = 0.209; Target contrast × Main axis of reported target: *F*(6,36) = 0.776, *p* = 0.526, *η^2^* = 0.115). The three-way interaction effect of all three factors was also not significant (*F*(12,72) = 0.756, *p* = 0.554, *η^2^* = 0.112).

**Reference**

Annis, R.C., and Frost, B. (1973). Human visual ecology and orientation anisotropies in acuity. Science 182, 729-731.





**Supplementary Figure 1. (A)** bootstrap distributions of the fitted *d_max_* under different conditions. The lines represent 90% confidence interval obtained from the distributions. **(B)** same as **(A)** but for the fitted contrast *c_50_*.





**Supplementary Figure 2. (A), (C), (E)** bootstrap distributions of the differences of the fitted *d_max_* between different conditions. The dashed lines represent 95% percentile of the distributions. The solid lines denote the empirically observed differences of the fitted *d_max_* between corresponding conditions. **(B), (D), (F)** bootstrap distributions of the differences of the fitted contrast *c_50_* between different conditions. The dashed lines represent 5% percentile of the distributions. The solid lines denote the empirically observed differences of the fitted contrast *c_50_* between corresponding conditions.





**Supplementary Figure 3.** Residual as a function of fitting exponent n. The exponent n which results in the smallest residual in the nonlinear, least-squares fitting procedure and is indicated by the dip of the curve is the best-fitting exponent.

**

**

**Supplementary Figure 4. (A)** Left column: contrast response functions for different pre-cueing conditions when the main axis of the reported target was vertical**.** Middle column: parameter estimates of threshold *c_50_* for trials with different pre-cues for each participant*.* Right column: same as the middle column but for parameter estimates of asymptote performance d_max._ **(B)** Same as **(A)**, but only including the trials in which the reported target was near horizontal.





**Supplementary Figure 5. (A)** mean reaction times (RTs) of trials in which the main axis of the reported target was vertical, plotted for different pre-cues as a function of contrast intensity. **(B)** same as **(A)** but only for trials in which the main axis of the reported target was horizontal.
